# Quality of life after surgical decompression for a space-occupying middle cerebral artery infarct: A cohort study

**DOI:** 10.1186/s12883-015-0407-0

**Published:** 2015-08-28

**Authors:** Tessa van Middelaar, Edo Richard, H. Bart van der Worp, Pepijn van den Munckhof, Pythia T. Nieuwkerk, Marieke C. Visser, Jan Stam, Paul J. Nederkoorn

**Affiliations:** Department of Neurology, Academic Medical Center (AMC) Amsterdam, Meibergdreef 9, 1105 AZ Amsterdam, The Netherlands; Department of Neurology, Radboud University Medical Center, Nijmegen, The Netherlands; Department of Neurology and Neurosurgery, Brain Center Rudolf Magnus, University Medical Center Utrecht (UMCU), Utrecht, The Netherlands; Department of Neurosurgery, Academic Medical Center (AMC) Amsterdam, Amsterdam, The Netherlands; Department of Medical Psychology, Academic Medical Center (AMC), Amsterdam, The Netherlands; Department of Neurology, VU University Medical Center (VUmc), Amsterdam, The Netherlands

## Abstract

**Background:**

In patients with a space-occupying middle cerebral artery (MCA) infarct surgical decompression reduces the risk of death, but increases the chance of survival with severe disability. We assessed quality of life (QoL), symptoms of depression, and caregiver burden at long-term follow-up.

**Methods:**

Patients treated in two academic centres between 2007 and 2012 were included. Follow-up was at least six months. Patients and caregivers were interviewed separately. QoL was assessed with a visual analogue scale and the 36-item Short-Form health survey (SF-36); depression with the Hospital Anxiety and Depression Scale; and caregiver burden with the Caregiver Strain Index.

**Results:**

Twenty five patients were enrolled, of whom seven had an infarct in the dominant hemisphere. After a median follow-up of 26 months (IQR 11–46) the median SF-36 mental component score was 54.4 (IQR 45–60), indicating a mental QoL comparable to that in the general population. The median SF-36 physical component score was 32.7 (IQR 22–38), indicating a worse physical QoL. Dominance of the hemisphere did not influence QoL. 79 % of patients and 65 % of caregivers would, in retrospect, again choose for surgery. 26 % of patients had signs of depression and 64 % of caregivers were substantially burdened in their daily life.

**Conclusions:**

Mental QoL after surgical decompression for space-occupying MCA infarct is comparable to that in the general population, whereas physical QoL is worse. Dominance of the hemisphere did not influence QoL. The majority of caregivers experience substantial burden. Most patients and caregivers stand by their decision for hemicraniectomy.

## Background

A large space-occupying infarct in the middle cerebral artery (MCA) territory is associated with high mortality as a result of transtentorial herniation [1]. This occurs particularly in relatively young patients (around the age of 60 years) [2]. A pooled analysis of three randomized controlled trials (RCTs) has demonstrated that in patients aged 18 to 60 years surgical decompression, consisting of a hemicraniectomy and duraplasty, dramatically reduces mortality if performed within 48 h of stroke onset [3–6]. However, this treatment is associated with a trend to survival with moderately severe to severe disability, defined as a score on the modified Rankin Scale (mRS) of 4 or 5 [3]. Many physicians think that most surviving patients have an unacceptable quality of life (QoL) [7]. Whether QoL is indeed unacceptable after decompressive surgery for a space-occupying infarct remains unknown. Another related question is whether QoL is worse in patients with an infarct in the dominant hemisphere. In addition to patient outcomes, the impact on the life of caregivers has not extensively been studied, even though they are confronted with the consequences every day [8].

In the current study, we aimed to assess the long-term QoL, symptoms of depression and caregiver burden in patients who underwent surgical decompression for a space-occupying infarct.

## Methods

### Study design and population

We performed a retrospective cohort study with prospective data collection. We contacted all patients, and their caregivers, who had undergone surgical decompression for space-occupying MCA infarct in the Academic Medical Centre in Amsterdam (AMC) and the University Medical Centre in Utrecht (UMCU) between October 2007 and March 2012. Patients with additional involvement of the anterior or posterior cerebral artery were not excluded. The inclusion period started after completion of the ‘Hemicraniectomy After Middle cerebral artery infarction with Life-threatening Edema Trial’ (HAMLET) [4]. Both AMC and UMCU had participated in HAMLET. We included patients, and their caregivers, who were at least six months after the surgical decompression, to assess QoL after recovery from the acute phase and the initial rehabilitation period. We assumed that after this timeframe the lives of most of the patients and caregivers would have settled, and long-term consequences of the infarct would be clear. The study was approved by the medical ethics committee of the Academic Medical Centre in Amsterdam and the University Medical Centre in Utrecht. All patients and caregivers who participated gave written informed consent. The study was conducted in accordance with the Declaration of Helsinki. We retrospectively collected data on age, gender, affected hemisphere, arterial territory of the stroke, score on the National Institutes of Health Stroke Scale (NIHSS) and the Glasgow Coma Scale before surgery.

Patients were interviewed by one of the investigators (TM) at their home. We aimed at interviewing both the patient and his/her caregiver, but allowed participation of either of them if this was not feasible. To avoid socially desirable answers, interviews were preferably held with only the interviewee (patient or caregiver) and interviewer present. When the patient was not able to complete the interview alone (e.g. because of aphasia), he/she was interviewed in the presence of the caregiver. We used a proxy-assessment by the caregiver, when no data on QoL could be obtained directly from the patient (e.g. in severe aphasia) [9]. When participants did not want a face-to-face interview, they were given the option to complete the questionnaires in writing.

### Outcome measures

QoL was assessed by patients and caregivers (proxy assessment) with two assessment instruments with complementary properties: a visual analogue scale (VAS) and the Medical Outcomes Study 36-item Short-Form health survey (SF-36) [10, 11]. Scores on the VAS and SF-36 ranged from zero to 100, with high scores indicating better QoL. Scores on the SF-36 are combined into eight subscales, are standardized and aggregated into a mental component score and a physical component score. The component scores were created by standardizing the scores on the subscales to the general Dutch population, followed by combining these into mental and physical scores using factor score coefficients per subscale, and finally transforming these to a scale with a mean of 50 (i.e. comparable results with the general population) and a standard deviation of 10 [11, 12]. We used the depression subscale of the Hospital Anxiety and Depression Scale (HADS) to evaluate symptoms of depression in patients and caregivers, with a score of eight or higher indicating depression [13]. Caregiver burden was assessed with the Caregiver Strain Index (CSI) and a VAS on QoL (of the caregiver) [14]. A score of 7 or higher on the CSI reflects a considerable level of stress in the life of the caregiver [15]. Patients and caregivers were also asked the question: ‘knowing what you know now, would you again decide for the surgical decompression?’ This question could only be answered with ‘yes’ or ‘no’, because we anticipated that in this dichotomized way there would be a relatively high barrier to say ‘no’. The number of patients that nevertheless did say ‘no’ is therefore important to be included in our conclusion.

To be able to compare our patient population to the population in the RCTs, we assessed functional outcome using the mRS [4–6]. We asked patients whether they are right or left-handed, to determine the dominant hemisphere. To examine if the results on QoL and depression were influenced by cognitive impairment, we administered the Mini-Mental State Examination (MMSE). We could not reliably administer the MMSE in aphasic patients [16].

### Statistical analysis

Dichotomous variables were presented as frequencies and continuous variables as mean (SD) or median (IQR, interquartile range). Z-scores were calculated to compare results on the SF-36 to the general Dutch population [11]. Analyses were performed with the Mann–Whitney *U* test and the Spearman’s rank correlation coefficient. Due to the relatively small sample size multivariate analyses with adjustment for potential confounders were not possible and all analyses were considered exploratory. Statistical analysis was done with the Statistical Package for Social Sciences version 21.0 for Windows (SPSS Inc., Chicago, IL, USA).

## Results

### Patients

Between October 2007 and March 2012, 45 patients underwent surgical decompression for a space-occupying MCA infarct in one of the two participating centres. Of these patients 11 (24 %) died during the follow-up period. All nine patients of whom the date of death is known died within the first month after surgery. Of the 34 surviving patients 25 (74 %) were included in the present study. Eight patients declined to participate and one patient could not be traced. Clinical characteristics at time of the surgical decompression did not differ between included and excluded patients (Table 1). Mean age of the 25 included patients was 48 years (SD 8.9). Two patients were older than 60 years (8 %). Twenty-three patients (92 %) had a job before the event and 21 (84 %) had a partner. Seven patients (28 %) had an infarct in the dominant hemisphere resulting in aphasia. The median duration of follow-up was 26 months (IQR 11–46).

**Table 1:** Patient characteristics

	Participating patients (*n* = 25)	Patients not participating (*n* = 9)	Patients deceased (*n* = 11)
Demographics			
Men	19/25 (76 %)	4/9 (44 %)	7/11 (64 %)
Age at stroke	48 (29–66)	47 (34–60)	48 (29–67)
Age ≤60 years at stroke	23/25 (92 %)	9/9 (100 %)	9/11 (82 %)
Neurological examination			
NIHSS score	20 (9–29)	22 (15–26)	22 (18–32)
Glasgow coma score	10 (6–15)	9 (3–13)	6 (5–15)
Territory of the infarct			
MCA only	17/25 (68 %)	6/9 (67 %)	9/11 (82 %)
Dominant hemisphere	7/25 (28 %)	2/9 (22 %)	5/11 (46 %)

Data are presented as number (%) or median (range)

Neurological examination was executed before surgical decompression

NIHSS indicates National Institutes of Health Stroke Scale; MCA, middle cerebral artery

In five of the 25 cases we could only interview the caregiver, because the patient had severe aphasia (*n* = 3) or declined participation (*n* = 2). In three cases no caregiver was available. In four out of the seven patients with an infarct in the dominant hemisphere we could interview both the patient and caregiver, in three patients only the caregiver could be interviewed. Of the 40 interviews, 31 (80 %) were executed with only the interviewee (patient or caregiver) and interviewer present. Two patients completed the questionnaires in writing. Of the 22 participating caregivers, 18 (82 %) were partners of the patients and four (18 %) were other family members.

### Quality of life

The median score on the VAS for QoL was 49.7 (IQR 20–67). Scores on the SF-36 subscales are presented in Table 2. The median mental component score of SF-36 was 54.4 (IQR, 45–60), indicating comparable results to the mental QoL of general population, which was standardized to a mean score of 50. The median physical component score was 32.7 (IQR 22–38), indicating a lower physical QoL than in the general population.

**Table 2:** Mean scores on SF-36 subscales

SF-36 subscale	Sample mean	Population mean	z-score
Physical functioning	26.4 (27.3)	83.0 (22.8)	−2.48
Role physical	32.0 (34.2)	76.4 (36.3)	−1.22
Bodily pain	63.4 (27.0)	74.9 (23.4)	−0.49
Social functioning	68.0 (30.6)	84.0 (22.4)	−0.71
Mental health	64.6 (20.6)	76.8 (17.4)	−0.70
Role emotional	86.7 (30.4)	82.3 (32.9)	0.13
Vitality	55.0 (18.1)	68.6 (19.3)	−0.70
General health	50.1 (27.1)	70.7 (20.7)	−1.00

Data are presented as mean (SD)

Sample mean indicates data from our study population and population mean data from the general Dutch population [11]

In the z-scores the general population is used as reference

A positive z-score indicates a better QoL in our population than in the general population

When comparing patients with an infarct in the dominant versus the non-dominant hemisphere, we found no significant difference in QoL assessed with the VAS (49 vs. 53 respectively, *p* = 0.80), mental component score (52 vs. 51, *p* = 0.79) or physical component score (38 vs. 29, *p* = 0.22). We found no significant correlation of age with QoL on the VAS (ρ 0.19, *p* = 0.37), mental component score (ρ 0.22, *p* = 0.30) or physical component score (ρ -0.35, *p* = 0.09).

### Additional outcomes

Five patients (26 %) and five caregivers (23 %) had a score ≥8 on the depression subscale of the HADS, indicative of depression. 14 caregivers (64 %) had a score ≥7 on the CSI, which indicates a considerable level of burden. The median score on the VAS assessing QoL of caregivers was 65.3 % (IQR 49–79). Fifteen of 19 patients (79 %) and 13 of 20 caregivers (65 %) answered the question ‘knowing what you know now, would you decide again for surgical decompression?’ positively. One patient and two caregivers did not want to answer the question. Retrospective agreement with the surgery was similar for patients with an infarct in the dominant and non-dominant hemisphere (75 % vs. 80 %), and for their caregivers (57 % vs. 69 %).

At follow-up 14 patients (56 %) had a good functional outcome (mRS ≤ 3). 13 patients (52 %) lived at home. The MMSE could not be administered in eight of the 20 interviewed patients, because of aphasia (*n* = 4), decline to answer (*n* = 3) or written questionnaire (*n* = 1). Patients, in whom the MMSE could be administered had mild cognitive impairment (median MMSE 28, IQR 26–30).

## Discussion

We found that in the chronic phase after surgical decompression for a space-occupying MCA infarct, mental QoL is comparable to that in the general population, whereas physical QoL is worse. Depression is relatively uncommon, but the majority of caregivers feels substantially burdened. The majority of patients and caregivers would in retrospect again decide for the surgery.

Just 33 % of physicians think that the majority of patients treated with surgical decompression for a space-occupying infarct achieve an acceptable QoL [7]. Our study refutes this assumption because patients generally had a good mental QoL, had a risk of depression that is comparable to the stroke population in general (26 % vs. 29 %) and because the majority agreed in retrospect with the treatment [17]. These results are also confirmed by a recent review showing a QoL which is only moderately decreased compared to the general population [8].

Only seven out of the current 25 patients (28 %) had an infarct in the dominant hemisphere, suggesting reluctance to perform the surgery in aphasic patients, which is in line with previous reports [8]. An international survey showed that treatment decision is influenced by the presence of aphasia in 47 % of the physicians, favouring the non-dominant hemisphere [18]. Our findings do not support a more conservative approach in patients with an infarct in the dominant hemisphere. The results however should be interpreted with caution due to the small numbers of patients. A previous cohort study in a broader stroke population showed comparable results with a lesion in the right hemisphere as a predictor for an unsatisfactory QoL one month after stroke [19]. An explanation to the apparent lack of influence of aphasia on QoL is the general underestimation of the impact of emotional and cognitive disability on QoL, which is more prevalent in patients with an infarct in the non-dominant hemisphere [20, 21].

Little attention to caregiver burden is paid in studies evaluating the effect of decompressive surgery in a space-occupying stroke [8]. In line with our expectations severe disability of the patients is accompanied by a substantial level of burden, a low QoL and relatively high depression rates in caregivers in comparison to the general Dutch population (23 % vs. 3 %) [22]. Appropriate practical and emotional support during the rehabilitation process could positively affect the caregiver-patient relationship and a delay in institutionalization [23].

### Limitations of the study

Our study has several limitations. Firstly, the relatively small sample size precludes strong conclusions, although the main research questions could be addressed. It also prohibited us from performing multivariate analyses and controlling for potential confounders. Secondly, the study could be subject to attrition bias, with 74 % of the surviving patients that agreed to participate in the study. We have no information on the QoL of the patients that did not want to participate or who deceased during the follow-up period. Our study population is however comparable to the population of the three major trials regarding mortality (24 % vs. 22 %) and good functional outcome (52 % vs. 56 %) [3]. Our study population is also approximately comparable to the general Dutch population with regard to socioeconomic status. In the general Dutch population aged 45 to 55 years old 86 % has a job in comparison to 84 % in our study population and 87 % has a partner in comparison to 92 % in our study population.[24] We therefore think that our findings, in combination with the previous findings in the systematic review, provide sufficient guidance for clinicians when discussing this treatment with their patients and caregivers [8].

## Conclusion

In contrast to physicians’ presumptions, patients who have survived surgical decompression for a space-occupying MCA infarct have a good mental quality of life. Physical quality of life is reduced in comparison to the general population, which is in accordance with a poor functional outcome. QoL was not influenced by dominance of the hemisphere. Depression among these patients is relatively uncommon. The high level of caregiver burden suggests that better guidance in the chronic phase after stroke may be of help. Most patients and caregivers would in retrospect again decide for surgical decompression. The results of the study can be used to better inform patients and caregivers in the acute phase.

## References

[1] Feigin VL, Lawes CM, Bennett DA, Barker-Collo SL, Parag V (2009). Worldwide stroke incidence and early case fatality reported in 56 population-based studies: a systematic review. Lancet Neurol.

[2] Krieger DW, Demchuk AM, Kasner SE, Jauss M, Hantson L (1999). Early clinical and radiological predictors of fatal brain swelling in ischemic stroke. Stroke.

[3] Cruz-Flores S, Berge E, Whittle IR (2012). Surgical decompression for cerebral oedema in acute ischaemic stroke. Cochrane Database Syst Rev.

[4] Hofmeijer J, Kappelle LJ, Algra A, Amelink GJ, van Gijn J, van der Worp HB (2009). Surgical decompression for space-occupying cerebral infarction (the Hemicraniectomy After Middle Cerebral Artery infarction with Life-threatening Edema Trial [HAMLET]): a multicentre, open, randomised trial. Lancet Neurol.

[5] Vahedi K, Vicaut E, Mateo J, Kurtz A, Orabi M, Guichard JP (2007). Sequential-design, multicenter, randomized, controlled trial of early decompressive craniectomy in malignant middle cerebral artery infarction (DECIMAL Trial). Stroke.

[6] Jüttler E, Schwab S, Schmiedek P, Unterberg A, Hennerici M, Woitzik J (2007). Decompressive Surgery for the Treatment of Malignant Infarction of the Middle Cerebral Artery (DESTINY): a randomized, controlled trial. Stroke.

[7] Schwarz S, Kühner C (2012). Prognosis and quality of life after decompressive hemicraniectomy: a nationwide survey in Germany on the attitudes held by doctors and nurses. Nervenarzt.

[8] van Middelaar T, Nederkoorn PJ, van der Worp HB, Stam J, Richard E (2015). Quality of life after surgical decompression for space-occupying middle cerebral artery infarction: Systematic review. Int J Stroke.

[9] Oczkowski C, O'Donnell M (2010). Reliability of proxy respondents for patients with stroke: a systematic review. J Stroke and Cerebrovasc Dis.

[10] de Boer AG, van Lanschot JJ, Stalmeier PF, van Sandick JW, Hulscher JB, de Haes JC (2004). Is a single-item visual analogue scale as valid, reliable and responsive as multi-item scales in measuring quality of life?. Qual Life Res.

[11] Aaronson NK, Muller M, Cohen PD, Essink-Bot ML, Fekkes M, Sanderman R (1998). Translation, validation, and norming of the Dutch language version of the SF-36 Health Survey in community and chronic disease populations. J Clin Epidemiol.

[12] Hobart JC, Williams LS, Moran K, Thompson AJ (2002). Quality of life measurement after stroke: uses and abuses of the SF-36. Stroke.

[13] Bjelland I, Dahl AA, Haug TT, Neckelmann D (2002). The validity of the Hospital Anxiety and Depression Scale. An updated literature review. J Psychosom Res.

[14] Post MW, Festen H, van de Port IG, Visser-Meily JM (2007). Reproducibility of the Caregiver Strain Index and the Caregiver Reaction Assessment in partners of stroke patients living in the Dutch community. Clin Rehabil.

[15] Robinson BC (1983). Validation of a Caregiver Strain Index. J Gerontol.

[16] Barnay JL, Wauquiez G, Bonnin-Koang HY, Anquetil C, Pérennou D, Piscicelli C (2014). Feasibility of the Cognitive Assessment scale for Stroke Patients (CASP) vs. MMSE and MoCA in aphasic left hemispheric stroke patients. Ann Phys Rehabil Med.

[17] Ayerbe L, Ayis S, Wolfe CD, Rudd AG (2013). Natural history, predictors and outcomes of depression after stroke: systematic review and meta-analysis. Br J Psychiatry.

[18] Neugebauer H, Creutzfeldt CJ, Hemphill JC, Heuschmann PU, Jüttler E (2014). DESTINY-S: attitudes of physicians toward disability and treatment in malignant MCA infarction. Neurocrit Care.

[19] Rachpukdee S, Howteerakul N, Suwannapong N, Tang-Aroonsin S (2013). Quality of life of stroke survivors: a 3-month follow-up study. J Stroke Cerebrovasc Dis.

[20] McKevitt C, Fudge N, Redfern J, Sheldenkar A, Crichton S, Rudd AR (2011). Self-reported long-term needs after stroke. Stroke.

[21] Castellanos-Pinedo F, Hernández-Pérez JM, Zurdo M, Rodríguez-Fúnez B, Hernández-Bayo JM, García-Fernández C (2011). Influence of premorbid psychopathology and lesion location on affective and behavioral disorders after ischemic stroke. J Neuropsychiatry Clin Neurosci.

[22] Bijl RV, Ravelli A, van Zessen G (1998). Prevalence of psychiatric disorder in the general population: results of The Netherlands Mental Health Survey and Incidence Study (NEMESIS). Soc Psychiatry Psychiatr Epidemiol.

[23] Ryynänen OP, Nousiainen P, Soini EJ, Tuominen S (2013). Efficacy of a multicomponent support programme for the caregivers of disabled persons: a randomised controlled study. Z Gerontol Geriatr.

[24] Kerncijfers. CBS. 2012. http://www.cbs.nl/nl-NL/menu/cijfers/kerncijfers/default.htm Accessed 10-04-2015.

